# Left ventricular diastolic dysfunction in non-myocardial disorders

**DOI:** 10.1093/ehjci/jeae209

**Published:** 2024-08-22

**Authors:** Otto A Smiseth, Tom Kai Ming Wang, Allan L Klein, Sherif F Nagueh

**Affiliations:** Division of Cardiovascular and Pulmonary Diseases, Institute for Surgical Research, Oslo University Hospital, University of Oslo, Rikshospitalet, PO Box 4950 Nydalen, NO-0424 Oslo, Norway; Section of Cardiovascular Imaging, Heart, Vascular and Thoracic Institute, Cleveland Clinic, OH, USA; Section of Cardiovascular Imaging, Heart, Vascular and Thoracic Institute, Cleveland Clinic, OH, USA; Methodist DeBakey Heart and Vascular Center, Houston, TX, USA

**Keywords:** atrial fibrillation, diastolic function, left ventricle, left bundle branch block, pericardial pressure, pulmonary hypertension

## Abstract

This article reviews and discusses non-myocardial disorders that represent diagnostic challenges when evaluating patients for suspected heart failure with preserved left ventricular ejection fraction. This includes pre-capillary pulmonary hypertension, which is important to differentiate from post-capillary hypertension caused by left-sided heart disease. The impact of electrical disorders on LV diastolic function is also reviewed, and includes a discussion of left bundle branch, which has both a direct effect on LV diastolic function, as well as a long-term effect due to remodelling. Furthermore, evaluation of diastolic function in patients with atrial fibrillation is discussed. Pericardial diseases are reviewed as well as effects of a normal pericardium on diastolic function in failing hearts. Finally, the article reviews how valvular diseases impact LV diastolic function.

## Graphical Abstract

**Graphical Abstract:**
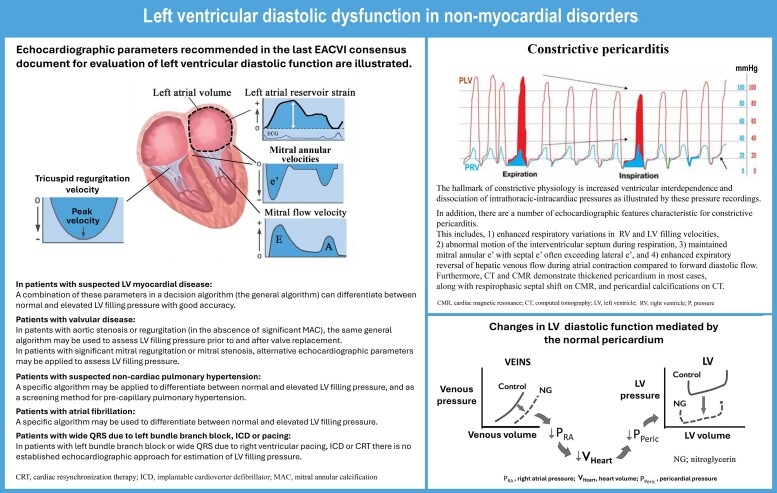
Upper left and upper right illustrations: Adapted from Smiseth *et al.*^3^ Lower right illustration: Adapted from Smiseth *et al.*^40^

This review discusses non-myocardial disorders that can influence left ventricular (LV) diastolic function as independent mechanisms or in combination with intrinsic myocardial dysfunction.

## Non-cardiac pulmonary hypertension

The most common aetiologies of pulmonary hypertension (PH) are LV dysfunction and mitral valve disease.^1^ Because elevated pulmonary artery pressure (PAP) in these cases reflects elevated left atrial (LA) and pulmonary venous pressures, the condition is named post-capillary PH. Less common is PH caused by elevated pulmonary vascular resistance, which is seen in patients with pulmonary vascular disease, pulmonary interstitial disease, or chronic thromboembolism and is named pre-capillary PH. In the latter case, LA pressure is normal unless there is simultaneous left-sided heart disease.

Since pre-capillary PH may require specific drug therapy that can be contraindicated for post-capillary PH, it is important to differentiate between the two conditions. The gold standard for diagnosing pre-capillary PH is right heart catheterization, and diagnostic criteria include elevated mean PAP (≥20 mmHg at rest) in the presence of normal LA pressure, measured indirectly as pulmonary capillary wedge pressure (PCWP) (≤15 mmHg).^1^

**Figure 1:**
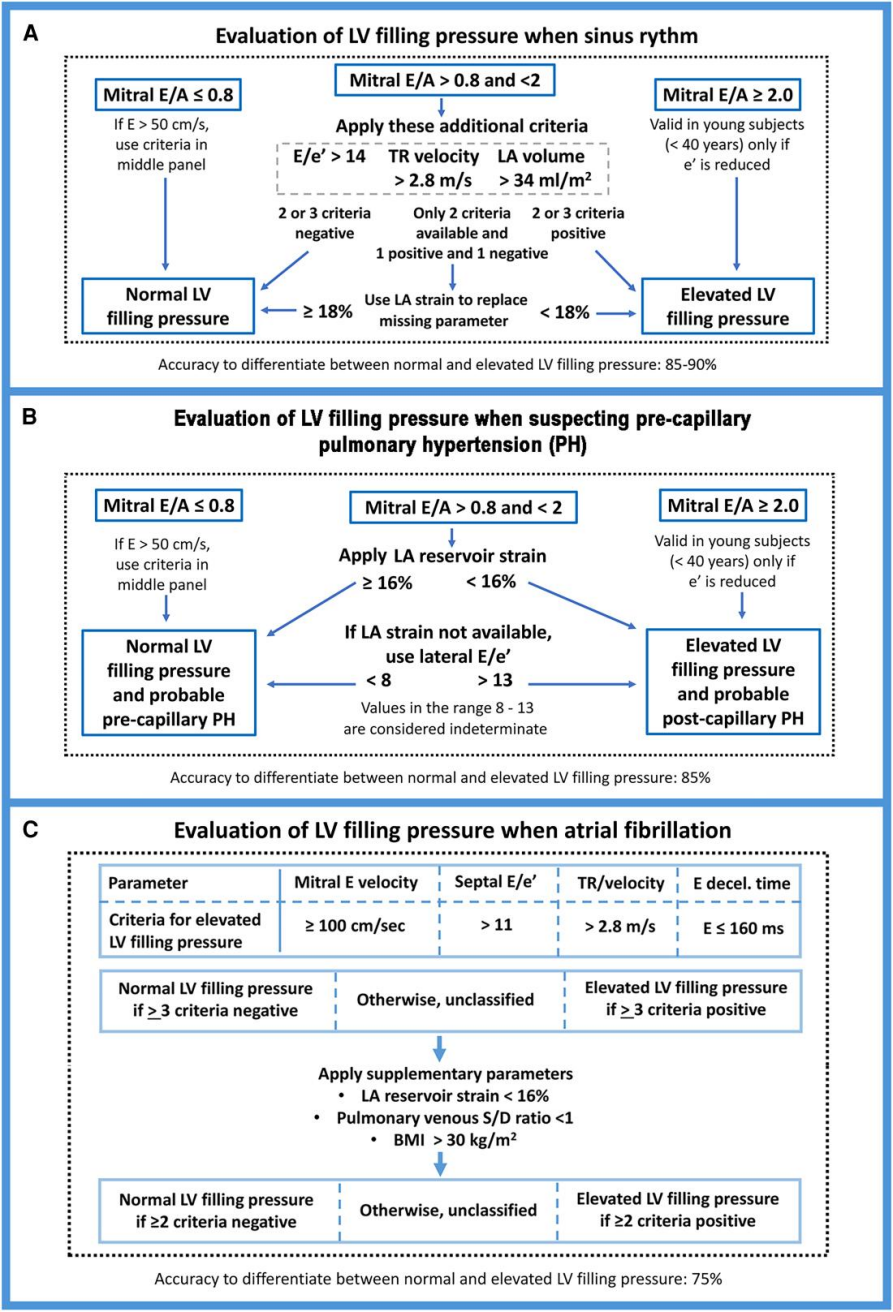
(*A*) Algorithm for estimation of LV filling pressure. To be used in patients in sinus rhythm but should not be applied in any of the following conditions: atrial fibrillation, LBBB/RV pacing/ICD, hypertrophic cardiomyopathy, severe mitral regurgitation, mitral stenosis, mitral annular calcification, mitral repair, or mitral prosthesis. Adapted from Smiseth *et al*.^3^ (*B*) Proposed algorithm for estimating LV filling pressure by echocardiography when suspecting pre-capillary pulmonary hypertension. Based on Inoue *et al*.^4^. PH, pulmonary hypertension; LV, left ventricular; LA, left atrial. (*C*) Proposed algorithm for estimating LV filling pressure in patients with atrial fibrillation. Adapted from Khan *et al*.^5^

Since there is limited access to right heart catheterization to make a conclusive diagnosis regarding pre-capillary PH, it would be useful with a non-invasive method to estimate LA pressure. An international consensus document recommends using a combination of echocardiographic parameters to differentiate between normal and elevated LA pressure.^2,3^  *Figure 1A* shows an algorithm that was recently recommended by the European Association on Cardiovascular Imaging.^3^ This algorithm, however, cannot be used to differentiate between pre- and post-capillary PH. This is because it uses peak tricuspid regurgitation (TR) velocity as one of the criteria, and because patients with pre-capillary PH often have abnormal septal motion that renders septal E/e′ less useful as marker of LV diastolic pressure. It was shown by Ruan and Nagueh^6^ that lateral E/e′ may be used as single parameter to differentiate between normal and elevated LA pressure. This works well for lateral E/e′ < 8 and >13, indicating normal and elevated LA pressure, respectively. In patients with lateral E/e′ in the range 8–13, however, this parameter could not differentiate between normal and elevated LA pressure, which is an important limitation since lateral E/e′ values in this range are common.

In a recent study in patients with unexplained dyspnoea suspected of heart failure or PH, it was shown that mitral E/A in combination with LA reservoir strain, differentiated between normal and elevated LV filling pressure with good accuracy of 85%.^4^ When restricting the analysis to patients with lateral E/e′ < 8 or >13, the accuracy to differentiate between normal and elevated LV filling pressure was similar (86%). Based upon these findings, a decision algorithm was suggested as illustrated in *Figure 1B*. The study of Inoue *et al*.^4^ was of only moderate size and single centre. However, the role of LA strain as a strong diagnostic marker to differentiate between normal and elevated LA pressure is supported by two larger multicentre studies.^7,8^

Importantly, right heart catheterization is needed when making therapeutic decisions in patients with pre-capillary PH. Furthermore, some patients have combined pre- and post-capillary PH and this diagnosis also requires right heart catheterization. Since echocardiography is readily available at low cost, it is well suited as a preliminary test in patients suspected of pre-capillary PH.

## Left bundle branch block

Left bundle branch block impacts LV diastolic function by different mechanisms. First, there is a direct effect on diastolic function because of the contractile dyssynchrony, as shown previously.^9^ Second, adverse LV remodelling that may occur in hearts with LBBB leads to slowing of LV relaxation and there may be compensatory elevation of LV filling pressure.^10^ Furthermore, patients with LBBB often have mitral regurgitation (MR).

**Figure 2:**
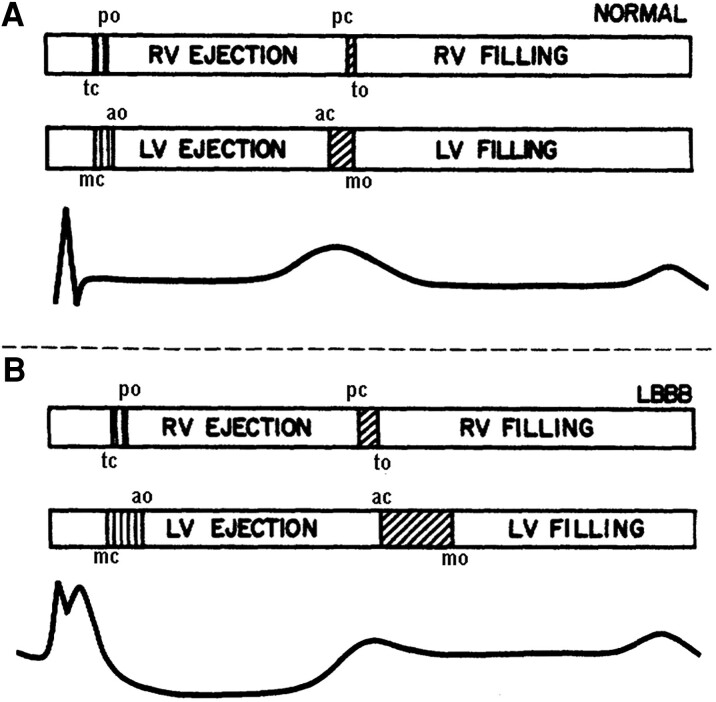
Abbreviated LV filling time in LBBB. The upper panel (*A*) is from a normal subject and the lower panel (*B*) from a patient with LBBB. The abbreviations po, to, ao, and mo refers to pulmonic, tricuspid, aortic, and mitral valve openings, respectively; pc, tc, ac, and mc refer to valve closures. The time interval from mc to ao represents the LV isovolumetric contraction time and the time from ac to mo the LV isovolumetric relaxation time, and both are prolonged in LBBB. Furthermore, there is prolongation of LV ejection time. The result is reduction in diastolic filling time. Modified from Grines *et al*.^11^

Because LBBB is associated with prolongation of the isovolumic contraction phase and slowing of myocardial relaxation, there is less time for LV diastolic filling.^11,12^ This is illustrated in *Figure 2* that compares diastolic filling times in the RV and LV.

Besides studies on filling time, there is limited insight into how LBBB modifies LV diastolic function. A non-invasive study^13^ suggested that LBBB was associated with elevated LV filling pressure. In another study that measured LV pressure, LBBB was associated with a moderate elevation of LV end-diastolic pressure, but the study was small and not conclusive.^12^

**Figure 3:**
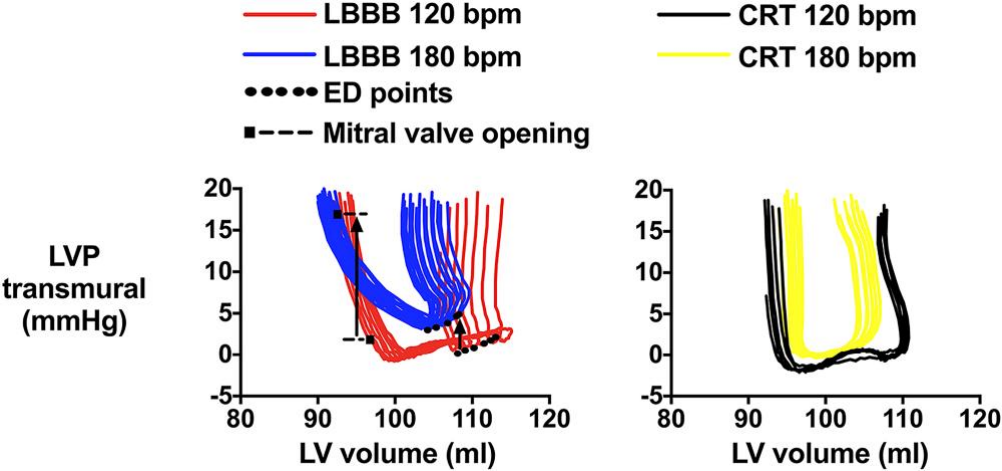
Effect of pacing tachycardia on LV diastolic stiffness: recordings from a representative experiment by Andersen *et al*.^9^ A series of pressure–volume loops were obtained by constriction of the caval veins. The left panel shows a distinct upward shift of the diastolic pressure–volume relationship by pacing tachycardia. As shown in the right panel, the pacing-induced upward shift was essentially abolished by CRT. bpm, beats/min; CRT, cardiac resynchronization therapy; ED, end-diastole; LVP, LV pressure. Modified from Andersen *et al*.^9^

A more recent experimental study showed that pacing tachycardia in hearts with LBBB caused marked elevation of mean LA pressure.^9^ The LV relaxation rate in hearts with LBBB was reduced compared with hearts with narrow QRS. During pacing tachycardia hearts with LBBB showed marked further slowing of global LV relaxation that caused increased LV diastolic stiffness, as indicated by an upward shift of the LV transmural pressure–volume relation, which is shown in *Figure 3*. As demonstrated by diastolic pressure-segment length analysis, the increased diastolic stiffness was attributed to delayed relaxation in the late-activated LV lateral wall. At baseline heart rate, LBBB had only minor impact on diastolic pressures, as there was sufficient time for complete relaxation. Furthermore, it was shown that CRT abolished the diastolic stiffening during tachycardia. It remains to be studied if a similar mechanism is operative in patients with LBBB.

Evaluation of LV diastolic function by echocardiography is challenging in hearts with LBBB, and there is currently no consensus on how to estimate LV filling pressure or evaluate grading of diastolic dysfunction in these patients. The accuracy of mitral annular velocities and E/e′ ratio is less in the presence of left bundle branch block, RV pacing, and in patients who have received cardiac resynchronization therapy.^2^ Therefore, the algorithm shown in *Figure 1A* should not be applied in patients with LBBB or other conditions with wide QRS such as patients receiving cardiac resynchronization therapy or right ventricular pacing.

## Atrial fibrillation

For a review of diastolic dysfunction in atrial fibrillation, it is referred to an article by B. Popescu *et al*. in this issue of the journal.^14^

Several different echocardiographic parameters have been proposed as markers of LV filling pressure in atrial fibrillation, but their validation is limited, and they have not been adopted in clinical routine.^15–18^ In a recent multicentre study, we investigated if a combination of multiple echocardiographic and clinical parameters may be used to evaluate LV filling pressure more effectively in atrial fibrillation.^5^ A main finding was that no single parameter had a sufficiently strong association with LV filling pressure to be recommended for clinical use. However, when combining several echocardiographic parameters and body mass index, it was possible to differentiate between patients with normal and elevated LV filling pressure with accuracy of 75%. The suggested algorithm is shown in *Figure 1C*. The feasibility of this approach was 85%.

Potentially, better standardization of measurements than in the study by Khan *et al*. may improve the diagnostic accuracy of echocardiography in the assessment of LV filling pressure in atrial fibrillation.

## Pericardial diseases

Constrictive pericarditis (CP) is an important differential diagnosis for HFpEF^19^ as patients typically also present with symptoms and signs of right HF and a low output state with a preserved LVEF. CP is caused by a number of disorders that increase pericardial stiffness due to pericardial thickening, inflammation and scarring, and there is often pericardial calcification. Echocardiography is the initial test of choice, with CMR or CT as adjunctive tests if echocardiography is non-diagnostic or if additional anatomic information is needed, such as the degree of pericardial thickness, inflammation, or calcification.^19^

**Figure 4:**
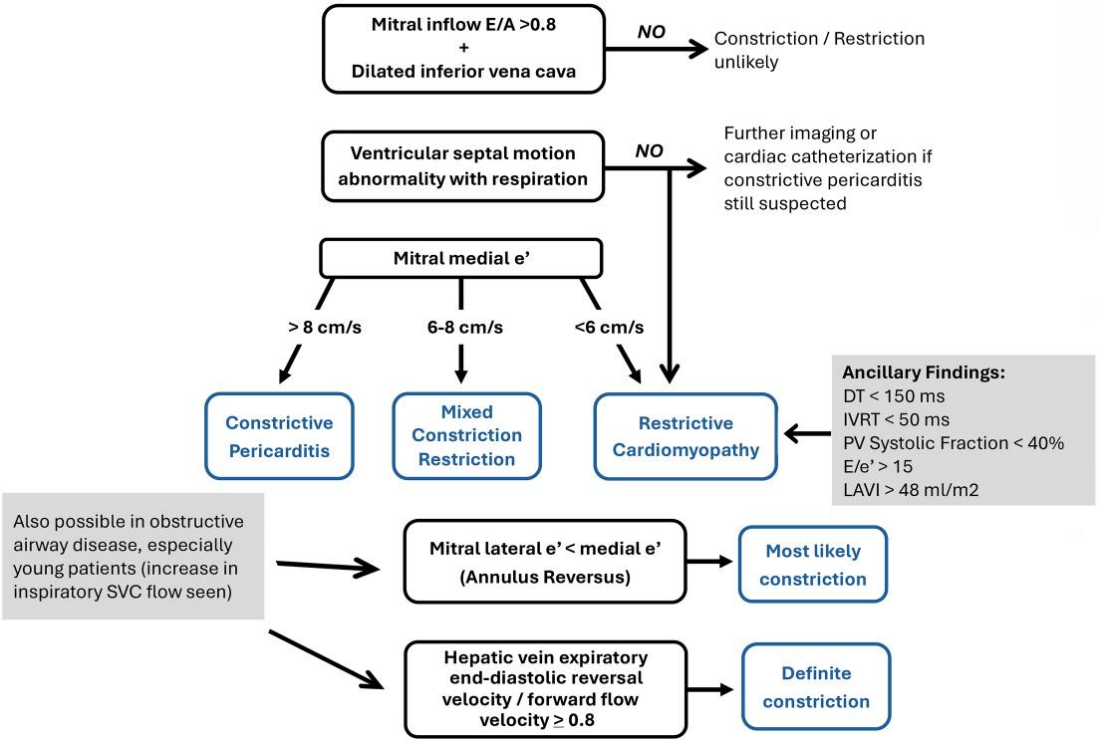
Algorithm for the diagnosis of constrictive pericarditis as well as comparison with restrictive cardiomyopathy. The figure is based on data from Syed *et al*.^20^ and Welch *et al*.^21^

Differentiation between CP and restrictive cardiomyopathy is challenging even when invasive data are available. As shown in the diagnostic algorithm in *Figure 4*, in CP, there is typically both a dilated vena cava and a mitral E/A > 0.8. In addition, there are a number of echocardiographic features characteristic for CP. This includes (i) enhanced respiratory variations in RV and LV filling velocities, (ii) abnormal motion of the interventricular septum during respiration, (iii) maintained mitral annular e′ with septal e′ often exceeding lateral e′, and (iv) enhanced expiratory reversal of hepatic venous flow during atrial contraction compared with forward diastolic flow. Furthermore, CT and CMR demonstrate thickened pericardium in most cases.

**Figure 5:**
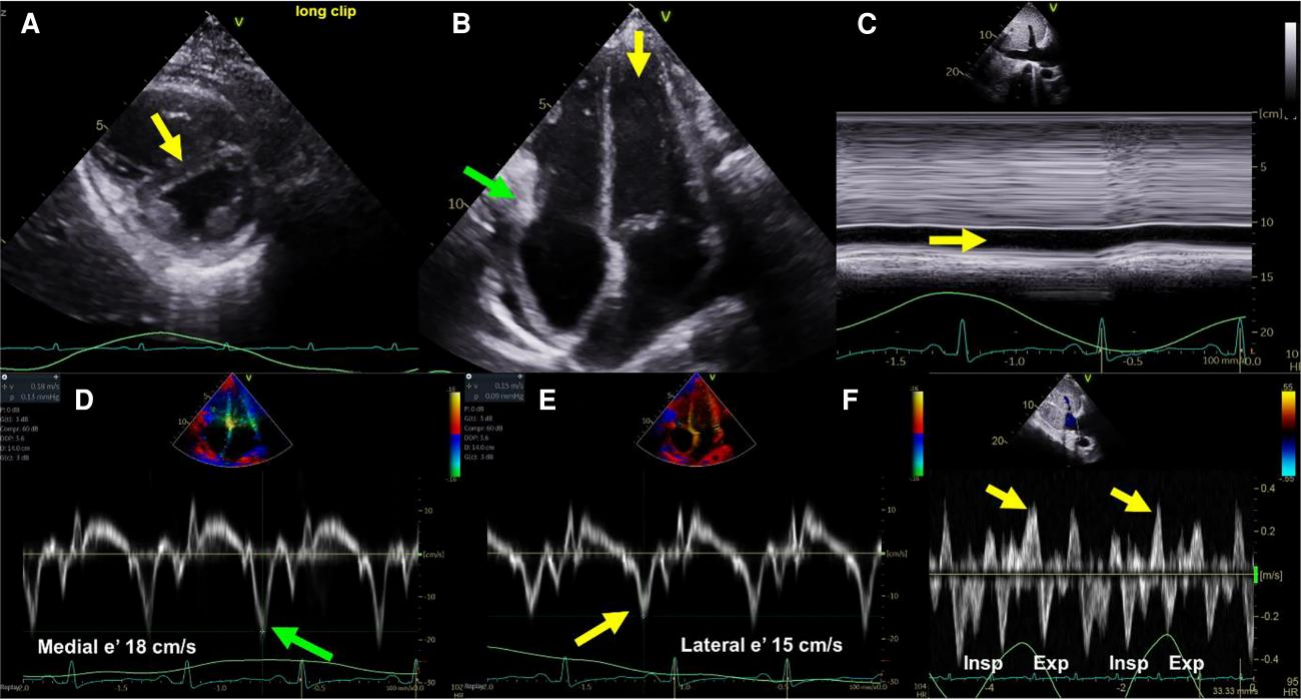
Echocardiography features of constrictive pericarditis. (*A*) Respirophasic interventricular septal motion abnormality, with leftward deviation and flattening of the septum during inspiration (yellow arrow). (*B*) Cylindrical deformity of the left ventricle (yellow arrow), and right ventricular lateral wall calcification and tethering (green arrow). (*C*) Inferior vena cava dilated (>2.1 cm) with minimal collapse <50 % with respiration. (*D*) and (*E*) shows mitral annular tissue Doppler e' velocity of 15 cm/s lateral (yellow arrow) and 18 cm/s medial (green arrow) indicating annulus reversus. (*F*) Increased end-diastolic reversal velocity of hepatic vein (yellow arrows) during expiration (>0.80 of forward velocity).^3^

The hallmark of constrictive physiology is increased ventricular interdependence and dissociation of intrathoracic–intracardiac pressures caused by a constricting pericardium. In contrast to a normal heart where inspiration leads to a small increase in peak tricuspid E and a small decrease in mitral E-velocities,^22^ CP is associated with marked increase in the tricuspid E velocity (>40%), and marked decrease (>25%) in mitral E velocity. *Figure 5F* shows typical expiratory reversal of end-diastolic flow within the hepatic veins in a patient with CP.

The enhanced respiratory variation in RV and LV filling and the abnormal septal motion in CP reflect both a restricted cardiac volume within the stiff pericardium and reduced transmission of respiratory changes in pleural pressure across the pericardium. To accommodate an increased RV volume during inspiration, the septum shifts to the left, causing reduction in LV volume. The leftward septal shift is enhanced by reduction in LV inflow due to reduced pressure gradient between the pulmonary veins and the left side of the heart. This is a reflection of reduced transmission of inspiratory fall in intrathoracic pressure to the left ventricle, whereas transmission to pulmonary vasculature is preserved. Expiration has the opposite effect as the septum shifts back towards the RV. This leads to reduced capacity for filling of the RV, and therefore expiration is associated with increased reversal of flow into the hepatic veins during right atrial contraction. The magnitude of the respiratory variations in filling velocities is often less marked, and the characteristic respiratory variation of the mitral inflow may not be seen in 30% of constrictive patients. Therefore, additional diagnostic criteria are needed to verify CP.

Typical findings in restrictive cardiomyopathy are short mitral E deceleration time, elevated E/e′, dilated atria, a plethoric inferior vena cava, and an abnormal pulmonary venous flow pattern (S/D <1).^21,23^ However, in contrast to diseases of the myocardium, where elevated LV filling pressure is associated with high E/e′, in patients with CP elevated LV filling pressure tends to be associated with low values of E/e′ (‘annulus paradoxus’). This is attributed to a ‘paradoxical’ increase in septal e′ (>8 cm/s) while the constrictive pathology progresses, resulting in a preserved or decreased septal E/e′ ratio.^19^

Tethering of the LV lateral and right ventricular free walls also contributes to the constrictive physiology and is demonstrated by an increased ratio of the septal to lateral mitral annular systolic velocities on tissue Doppler imaging (‘annulus reversus’) (*Figure 5D* and *E*). Similarly, the lateral LV and RV free wall peak systolic strain is diminished when compared with the septal peak systolic strain (‘strain reversus’).^19^ Important caveat to note is the influence of primary constriction and mixed constriction/restriction on the haemodynamics as well as septal annular velocities.^24,25^ With mixed constriction/restriction as in radiation heart disease, the septal annulus e′ velocity may not be increased, at 6–8 cm/s, *Figure 4*. The septal e′ velocity may be the best prognostic parameter for long-term outcome after pericardiectomy.^26^ It should also be emphasized that one-third of the patients with constriction may present with TR that could impact patients’ prognosis and often tricuspid repair may be warranted during pericardiectomy.

Other imaging techniques, such as cardiac gated CT and CMR, have emerged as important tools when faced with diagnostic uncertainty in separating CP from restriction.^27^ Aside from establishing constrictive haemodynamics, CMR can characterize the pericardium, prognosticate, and guide therapy.^28^ A thickened pericardium, a high pericardial signal on T2 weighted images and late gadolinium enhancement acquisitions, representing abnormal vascular permeability and oedema, indicates active pericardial inflammation and should prompt a trial of anti-inflammatory medication to reverse the constrictive physiology (‘transient CP’). Cardiac CT offers accurate delineation of pericardial thickening and calcification, confirming the diagnosis and is useful in pre-operative planning, particularly in the setting of previous open-heart surgery.

**Figure 6:**
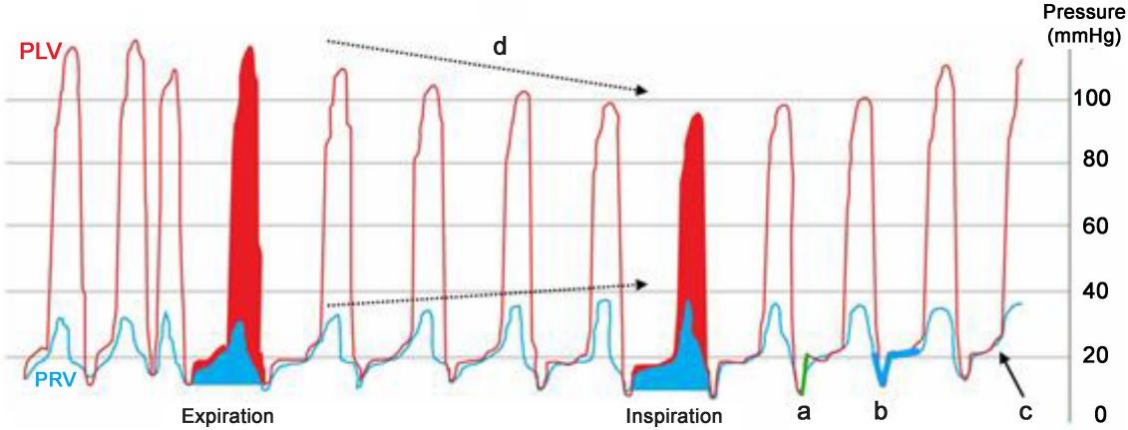
Simultaneous RV (blue tracing) and LV (red tracing) pressure tracings from an invasive haemodynamic study during cardiac catheterization. Both ventricles demonstrate a rapid early-diastolic rise in pressure > 7 mmHg (*A*), diastolic filling demonstrating the square root pattern (*B*), and equalization of RV and rise in late-diastolic pressures (<5 mmHg) (*C*). There is ventricular interdependence (*D*) and a systolic area index ([RV area/LV area on inspiration]/[RV area/LV area on expiration]) of 1.2 (blue/red tracings). The findings are suggestive of constrictive pericarditis. Illustration by AL Klein in Smiseth *et al*.^3^

When initial imaging is non-diagnostic and clinical suspicion lingers, right and left heart catheterization with haemodynamic assessment is the next step to confirm constrictive physiology (*Figure 6*). The presence of a rapid filling wave more than 7 mmHg (*Figure 6A*) coupled with near equalization of the LV and RV end-diastolic pressures (*Figure 6C*), is suggestive of constrictive physiology. The most salient finding however, is demonstrating LV/RV interdependence, best shown by decreasing LV systolic and increasing RV systolic pressures on inspiration (*Figure 6D*).^20^

Ultimately, when the disease progresses and patients become symptomatic, radical pericardiectomy is warranted, a procedure that can reverse most of the salient findings of constrictive physiology.

In summary, in the setting of clinical right heart failure, the presence of preserved or increased septal e′ (>8 cm/s), once high output failure is excluded, with associated ventricular interdependence suggested by a respirophasic septal shift, and/or end-diastolic expiratory flow reversal within the hepatic veins, can be used to diagnose CP (*Figure 1*). Cardiac CT can accurately assess pericardial calcium burden, while pericardial characterization using CMR identifies active inflammation in transient inflammatory constriction and can guide medical management.

## Extraventricular pressure

In clinical routine, LV end-diastolic pressure or pulmonary capillary wedge pressure is used to represent LV filling pressure and preload. Theoretically, a more appropriate measure of LV preload is the transmural filling pressure that is calculated as LV pressure minus pericardial pressure. In most clinical situations, there are concordant changes in LV intracavitary and transmural pressure. However, as discussed in this section, vasoactive agents may cause marked changes in LV end-diastolic pressure, which do not represent changes in preload. Furthermore, when there is elevated intrathoracic pressure, such as during mechanical ventilation with positive end-expiratory pressure (PEEP) ventilation, there is typically elevated LV intracavitary pressure, whereas transmural filling pressure is reduced. It is referred to a recent review by Pinsky^29^ on the physiology and clinical impact of cardiopulmonary interactions, and to a comprehensive review by Borlaug and Reddy^30^ on the role of the normal pericardium for cardiac function. This section focuses on how changes in LV extraventricular pressure may impact LV diastolic function and the interpretation of the measured LV diastolic pressures.

In principle, the different cardiac chambers are competing about a limited space within the pericardium, which is normally a stiff structure. Therefore, when one chamber dilates, e.g. the relatively compliant RV, the pericardium is stretched and there is less space for the LV. The resulting elevation of pericardial pressure acts as a compressive force on the LV and therefore LV diastolic pressure becomes elevated, whereas LV end-diastolic volume may decrease. In congestive heart failure with enlarged heart and stretched pericardium, the strength of ventricular interactions mediated by pericardium tends to be enhanced.

Following a study by Alderman and Glantz^31^ and other studies that observed acute shifts of the LV diastolic pressure–volume relationship during administration of vasoactive agents, there was a strong interest in understanding mechanisms of these changes in diastolic function. John Tyberg proposed the ‘pericardial hypotheses’, i.e. that the pressure–volume shifts were caused by unappreciated changes in pericardial pressure,^32^ rather than by a change in myocardial compliance.

**Figure 7:**
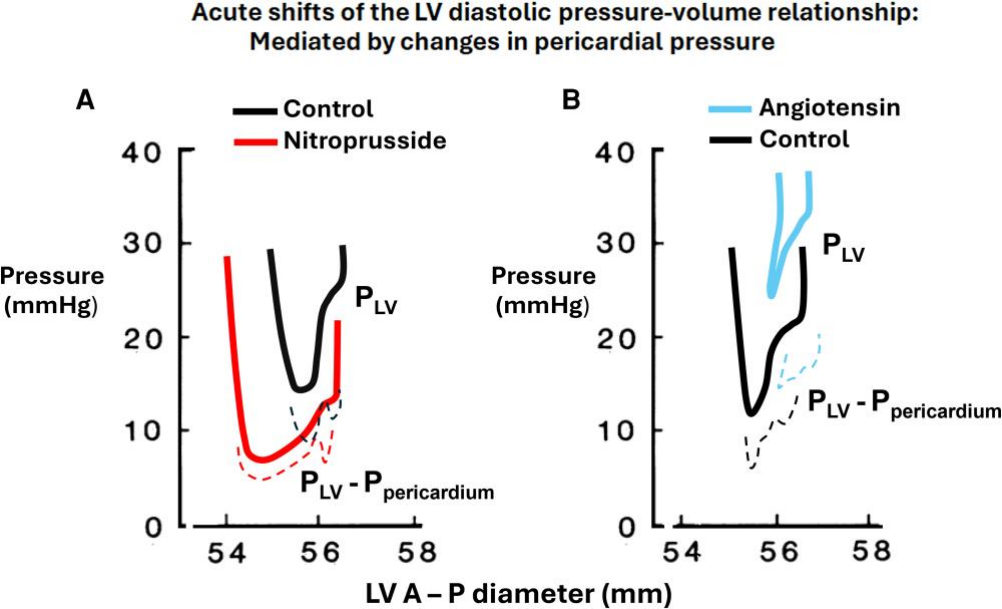
Effects of nitroprusside and angiotensin on the left ventricular diastolic pressure–diameter relationship in a dog with markedly elevated LVEDP. Sodium nitroprusside shifted the left ventricular diastolic pressure–diameter relationship downwards, (*A*) whereas angiotensin caused an upward shift. The left ventricular transmural pressure–diameter relationship (dotted lines) showed only minor changes. (*B*) Therefore, the pressure–diameter shifts with these agents were attributed to changes in pericardial pressure. Modified from Smiseth *et al*.^34^

A first step in exploration of this hypothesis was establishment of methodology to measure pericardial constraint. As shown in experimental studies,^33^ this was feasible by introducing a flat fluid-containing balloon into the pericardial space to measure the force (normal stress) exerted by the parietal pericardium on the surface of the heart. *Figure 7* is from a study that measured pericardial pressure by the flat balloon method. This experimental study reproduced the clinical observations that vasoactive drugs could shift the LV diastolic pressure–volume curves.^34^ The vasodilator drug sodium nitroprusside and the vasoconstrictor angiotensin shifted the LV diastolic pressure–volume curve downwards and upwards, respectively. When adjusting for changes in pericardial pressure by using the LV transmural pressure–volume relationship to reflect myocardial stiffness, there was no apparent shift of the diastolic pressure–volume curves with these vasoactive agents, consistent with unchanged myocardial stiffness.

**Figure 8:**
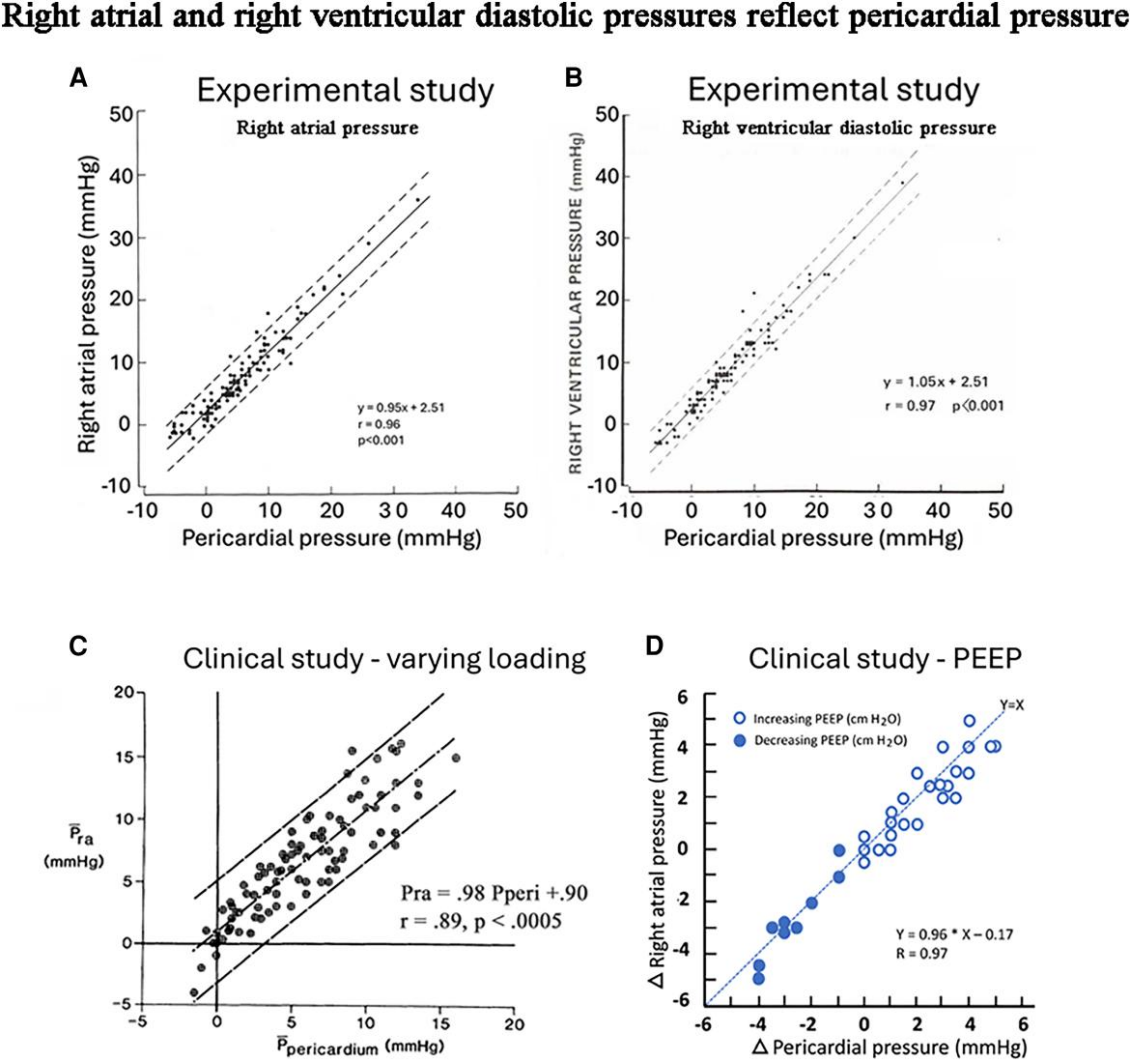
Right atrial and RV diastolic pressure approximate pericardial pressure: (*A* and *B*) data from an experimental study in acute heart failure showing a strong association between RA and RV mean diastolic pressure and pericardial pressure. From Smiseth *et al*.^35^ (*C*) An intraoperative, clinical study showing a strong association between mean RA pressure and pericardial pressure. From Tyberg *et al*.^36^ (*D*) Clinical study showing that changes in mean right atrial pressure reflect changes pericardial pressure during positive pressure ventilation. From a study in eight patients in the early phase after heart surgery. From Smiseth *et al*.^37^ All measurements of pericardial pressure shown in panels *A*–*D* were done with flat liquid-containing balloon transducers.

**Figure 9:**
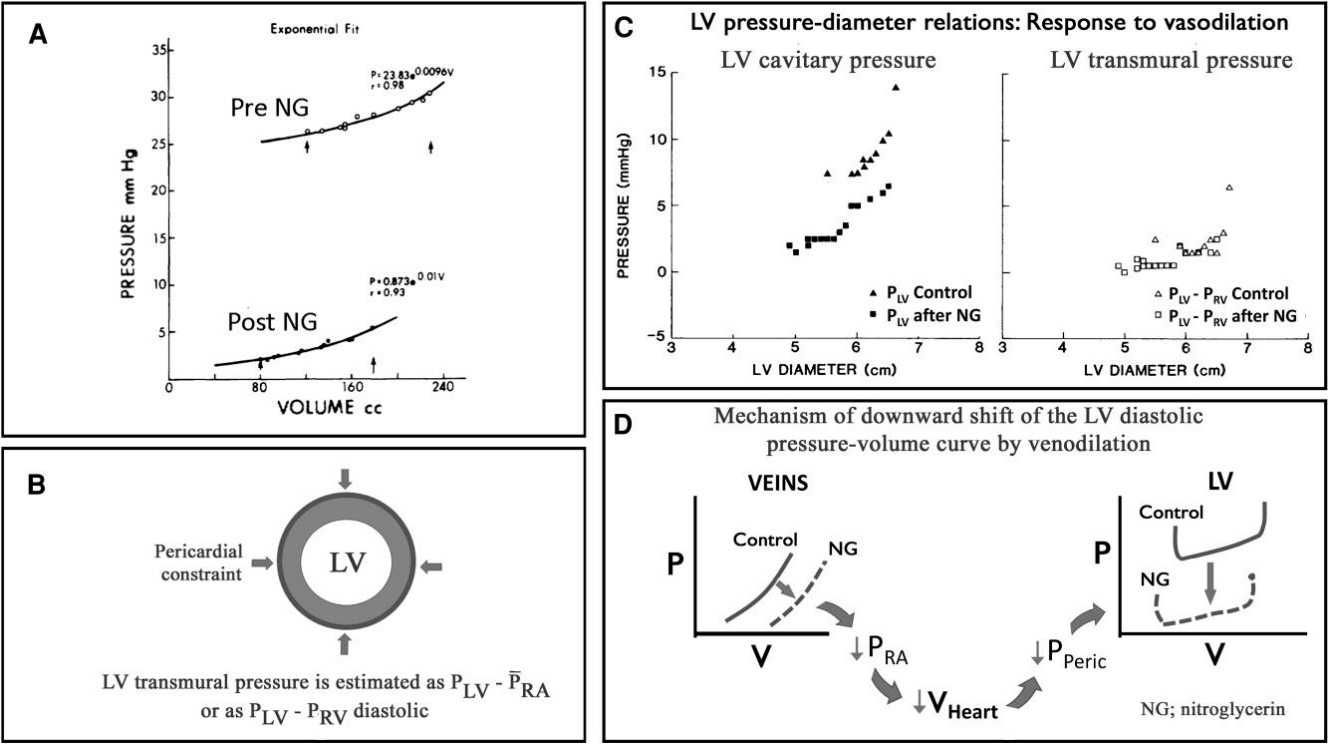
Panel *A* shows recordings from a patient with heart failure. Following intravenous nitroglycerine (NG), there is a marked downward shift of the LV diastolic pressure–volume relationship.^38^ Panel *B* shows calculation of LV transmural pressure, using mean RA pressure or RV diastolic pressure to represent pericardial pressure; Panel *C* shows LV cavitary pressure vs. diameter from a patient during heart catheterization (left).^39^ Note that the pressure–diameter coordinates are shifted downward after sublingual nitroglycerine. The LV transmural pressure–diameter relationship (right), however, did not shift, consistent with unchanged myocardial compliance. Panel *D* illustrates schematically how venodilation lowers intracardiac pressures and pericardial pressure, resulting in downward shift of the LV diastolic P–V relationship. Adapted from Smiseth et al.^40^.

An important additional observation made by Tyberg and Smiseth was that mean right atrial pressure (PRA) and right ventricular diastolic pressure approximate pericardial pressure.^35,36^ This was demonstrated first in an animal model and later in patients as shown in *Figure 8*. By using mean RA pressure as estimate of pericardial pressure, it was possible to calculate LV transmural pressure as PLV–PRA, and to calculate LV transmural pressure–volume curves. As shown in *Figure 9C*, this in turn could be used to differentiate between diastolic pressure–volume shifts caused by changes in myocardial stiffness, as reflected in the LV transmural pressure–volume relationship, as opposed to those caused by changes in pericardial pressure.^37^ The contribution that pericardial pressure makes to LV end-diastolic pressure is substantial and as shown in an intraoperative study may represent 40–50% of LV diastolic pressure.^36^ The relative contribution of pericardial pressure to LV end-diastolic pressure in chronic heart failure remains to be studied. In the absence of data on directly measured pericardial pressure, mean RA pressure may be used as an approximation of pericardial pressure. As shown in *Figure 8*, there is as expected, a small offset in the relationship between RA pressure and pericardial pressure, which means that only a small RA transmural pressure is needed to maintain RA volume. Importantly, in patients with RV and RA hypertrophy, RA transmural pressure will be higher and RA pressure will overestimate pericardial pressure.

In the next series of experiments, the Tyberg group showed how translocations of blood between the heart and the capacitance circulation could account for changes in cardiac chamber size and hence changes in pericardial pressure during administration of vasoactive drugs. They focused on the splanchnic vascular bed that represents the most important blood reservoir,^34^ and quantified venous capacity and compliance by construction of vascular pressure–volume curves.^34,41,42^ These principles are illustrated in *Figure 9D*.

A functional implication of downward shifts of the LV diastolic pressure–volume relation in failing hearts, with a typical case in *Figure 9A*, is that diastolic pressure and hence pulmonary venous pressure can be markedly reduced with minimal reduction in end-diastolic volume and preload. This mechanism contributes to maintaining LV stroke volume following administration of vasodilators and comes in addition to the beneficial effect of afterload reduction.

Presumably, physical exercise can shift the LV diastolic pressure–volume curves by a similar mechanism, but there are limited data on the topic. A recent study in patients with HFpEF demonstrated upward shifts of the LV diastolic pressure–volume curve during exercise, and the authors concluded that this was due to LV diastolic stiffening.^43^ Potentially, elevation of pericardial pressure contributed to the exercise-induced upward shifts. It would be interesting to do a similar protocol with measurement of RA pressure in addition to LV pressure during exercise for construction of LV transmural pressure–volume curves as a means to differentiate between a pericardial effect and myocardial stiffening.

**Figure 10:**
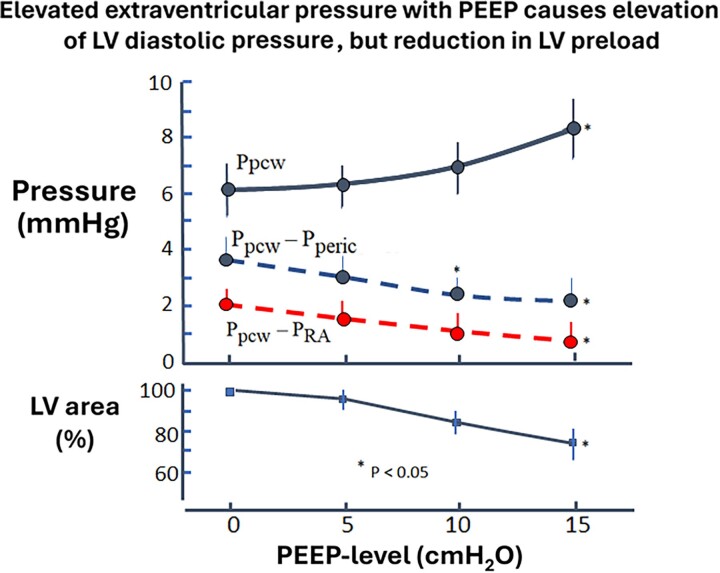
Effect of positive end-expiratory pressure (PEEP) ventilation on pulmonary capillary wedge pressure (Ppcw), transmural filling pressure, and left ventricular (LV) cross-sectional area. Data are mean value ± SEM. Left ventricular cross-sectional area is expressed as percent of the value at zero PEEP ventilation. Incremental PEEP increased pulmonary capillary wedge pressure and caused a progressive reduction in transmural filling pressure [pulmonary capillary wedge pressure minus pericardial pressure (Pperic) measured by fluid-containing flat balloon]. There was a parallel reduction in the estimated transmural filling pressure [pulmonary capillary wedge pressure minus right atrial pressure (Pra)] and in the normalized cross-sectional area. Data from six patients.^37^

Like effects of pericardial restraint on LV diastolic pressure, mechanical ventilation with PEEP that ultimately causes elevation of pericardial pressure also leads to upward shifts of the LV diastolic pressure–volume relations. This interaction is illustrated in *Figure 10*, showing recording taken following heart surgery.^37^ Increasing levels of PEEP caused increasing pulmonary artery wedge pressure, whereas both LV transmural filling pressure and LV cross-sectional area decreased, consistent with reductions in LV preload. Therefore, LV intracavitary filling pressure provided incorrect preload information. This is because increased pressure on the outside of the heart (pleural pressure and pericardial pressure) compresses the left ventricle so that the effective filling pressure is reduced. The effective filling pressure or transmural pressure is then a better measure of preload. The clinical utility of using calculated LV transmural filling pressure (by subtracting mean RA pressure) as a clinical measure when monitoring patients on PEEP remains to be studied.

## Estimation of LV filling pressures in patients with valvular heart disease

### Aortic stenosis

Patient with moderate or severe aortic stenosis (AS) can have impaired LV relaxation due to the increased LV systolic pressures due to AS. Further, with the development of LV hypertrophy and the increased interstitial fibrosis, LV chamber and myocardial stiffness are increased. Aside from these considerations, older patients with AS can have coexisting diseases that cause diastolic dysfunction like hypertension and cardiac amyloidosis. Depending on the intravascular volume status and LA function, LV diastolic pressures and LA pressures can be normal or increased. Therefore, the presence of normal LV filling pressures in AS patients does not mean they have normal LV diastolic function. Importantly, AS patients with diastolic dysfunction are at an increased risk for adverse cardiovascular outcomes, including heart failure.^44^

LV diastolic dysfunction can improve after surgical or transcatheter aortic valve replacement. This occurs due to improved LV relaxation with the decrease in LV systolic pressure. However, myocardial stiffness is increased in the early phase after valve surgery. This occurs despite the regression of myocyte hypertrophy, as interstitial fibrosis takes a much longer time to regress.^45^ Importantly, in the current era, where many patients with AS are treated with transcatheter aortic valve replacement, the improvement of LV diastolic function is associated with improved survival.^46,47^

Echocardiographic assessment of diastolic function in patients with AS is similar to patients without AS, and the algorithm shown in *Figure 1A* can be applied to patients with AS. The interpreting physician needs to look at quality of the acquired signals, the heart rhythm (sinus or atrial fibrillation), the presence of LBBB or RV pacing, and whether there is moderate or severe mitral annular calcification (MAC). If significant MAC is present, mitral E/A ratio and isovolumic relaxation time (IVRT) should be relied on to estimate LV filling pressures as opposed to E/e′ ratio. A recent study showed that LV filling pressure estimated by these variables in patients with significant AS and moderate or severe MAC is associated with outcome events.^48^ After aortic valve replacement, the algorithm shown in *Figure 1A* can be applied in the presence of sinus rhythm and in the absence of significant MAC.^49^

### Aortic regurgitation

Patients with chronic severe aortic regurgitation (AR) can have increased LV end-diastolic wall stress. This is related to the increased LV end-diastolic dimension that occurs usually in the presence of eccentric LV hypertrophy with normal or reduced wall thickness. Patients also develop increased interstitial fibrosis. As a result, LV chamber stiffness is increased. The increased interstitial fibrosis takes a long time to regress after aortic valve replacement and as a result, LV filling pressures can remain abnormally elevated despite successful valve replacement.^50^

There is a paucity of data looking at the accuracy of echocardiographic assessment of LV diastolic function in patients with AR. In the absence of coexisting significant mitral valve disease or MAC, the general algorithm shown in *Figure 1A* can be applied to estimate LV filling pressures in patients with significant AR. It can also be applied after aortic valve replacement to estimate LV filling pressures in these patients.^49^

### Mitral stenosis

The majority of patients with rheumatic mitral stenosis does not have LV diastolic dysfunction. However, LA pressure is elevated due to mitral stenosis. Older patients with calcific mitral stenosis can have diastolic dysfunction due to coexisting hypertension. The invasive evaluation of LV diastolic function in these patients relies on measurement of LV diastolic pressures.

Non-invasive estimation of LA pressure in patients with mitral stenosis is challenging. There are several findings in the mitral inflow pattern that can indicate the presence of elevated LA pressure. Patients with increased LA pressure usually have increased LA pressure into late diastole with increased peak mitral A velocity (>1.5 m/s). IVRT is short with earlier opening of the mitral valve due to increased LA pressure, whereas mitral E velocity is elevated, and E/A ratio is >1.^2^

With impaired LV relaxation, e′ velocity is delayed such that it occurs at the LA–LV pressure crossover point. In comparison, mitral E velocity occurs earlier with elevated left atrial pressure (LAP), and in this setting, an abbreviated time interval between onset mitral E and onset e′ (T_E–e′_) reflects elevated LA pressure. It was observed that the ratio IVRT/T_E–e′_ correlates well with mean PCWP and LAP in patients with mitral stenosis.^51^

### Mitral regurgitation

In patients with LV systolic dysfunction and secondary MR, several indices can be applied to draw inferences about mean LA pressure. This includes mitral E/A ratio, IVRT, deceleration time of mitral E, peak TR velocity, and average E/e′ ratio.^2^ The algorithm in *Figure 1A* may be applied to these patients.

**Figure 11:**
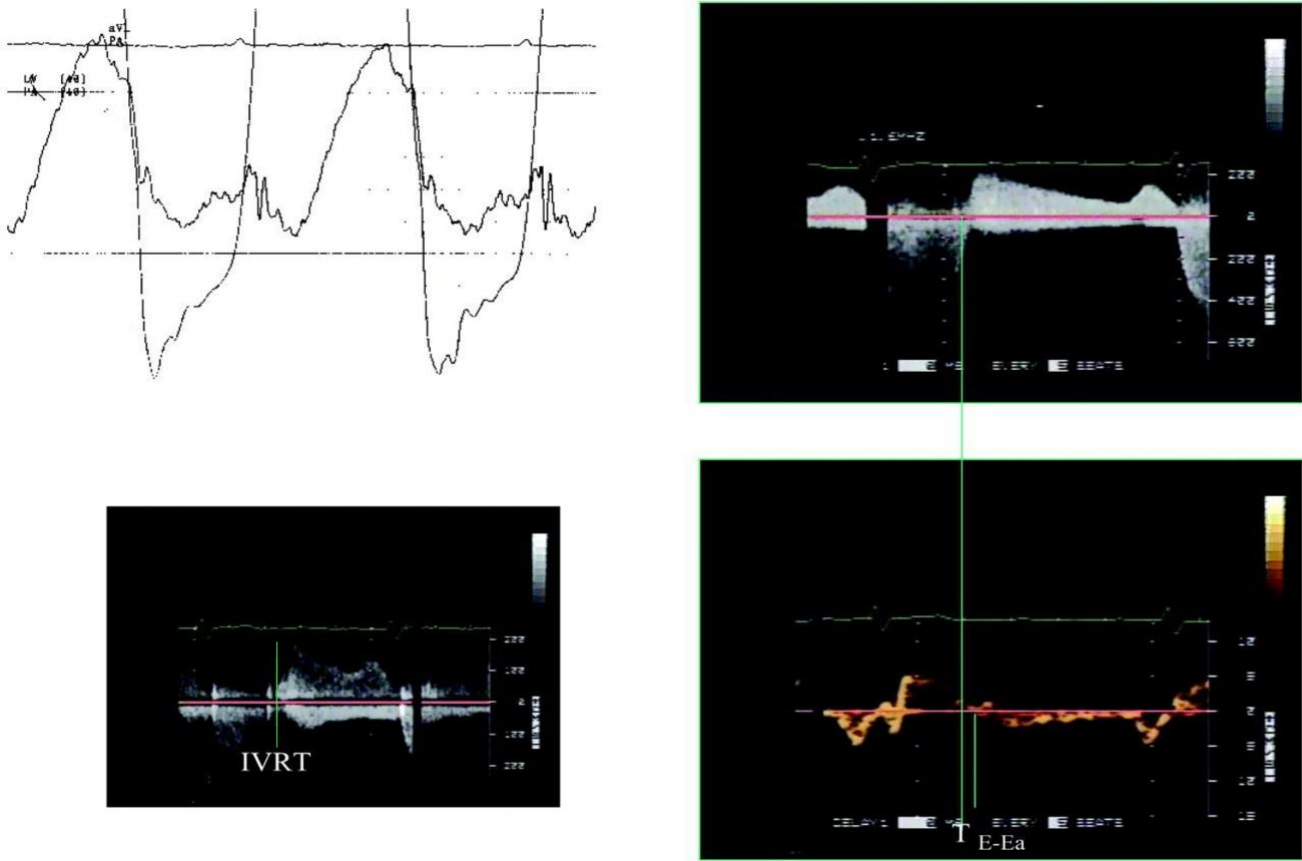
Pressure and Doppler signals from patient with combined mitral regurgitation and mitral stenosis. Upper left, LAP and LV pressure tracings; lower line marks 20 mmHg, and upper line, 40 mmHg. Upper right, mitral inflow. Lower left, IVRT (interval between end of aortic flow at closing click and green line marking onset of mitral inflow). Lower right, mitral annulus tissue Doppler recording at septal site of mitral annulus. Note prominent v wave in LAP. Mean LAP was 31 mmHg. Mitral valve area was calculated at 1 cm^2^. IVRT was 56 ms, and T_E–e′_ was 56 ms, giving an IVRT/T_E–e′_ ratio of 1. Ea in the lower right panel represents e′. Modified from Diwan *et al*.^51^

Mitral E/e′ ratio is not reliable for the estimation of mean LA pressure in patients with primary MR and normal LV EF as it can fall in the normal range despite elevated LA pressure.^51^ An alternative approach is using the ratio between IVRT and T_E–e′_ that correlates well with mean PCWP and LAP in patients with mitral regurgitation. *Figure 11* is from the study by Diwan *et al*.^51^ that showed how IVRT/T_E–e′_ may be used to assess LA pressure in patients with mitral valve disease.

It is also possible to draw inferences about mean LA pressure based on peak TR velocity and pulmonary arterial systolic pressure. However, in the presence of incomplete TR jet, ultrasound enhancing agent or agitated saline should be administered intravenously to allow for the recording of a complete TR jet.

## Data Availability

There are no new data generated in this paper.
